# *Alignoth*: portable and interactive visualization of read alignments

**DOI:** 10.1093/bioinformatics/btaf663

**Published:** 2025-12-14

**Authors:** Felix Wiegand, Felix Mölder, Johannes Köster

**Affiliations:** Bioinformatics and Computational Oncology, Institute for AI in Medicine (IKIM), University Hospital Essen, University of Duisburg-Essen, Essen 45131, Germany; Bioinformatics and Computational Oncology, Institute for AI in Medicine (IKIM), University Hospital Essen, University of Duisburg-Essen, Essen 45131, Germany; Institute of Pathology, University Hospital Essen, University of Duisburg-Essen, Essen 45147, Germany; Bioinformatics and Computational Oncology, Institute for AI in Medicine (IKIM), University Hospital Essen, University of Duisburg-Essen, Essen 45131, Germany

## Abstract

**Summary:**

We present Alignoth, a lightweight command line application that generates self-contained portable HTML reports of DNA sequencing read alignment pileups and additionally supports export to static formats such as PNG, SVG, and PDF as well as a JSON based embeddable representation. The HTML reports feature read name search and mapping-quality-based read highlighting, and require only minimal storage, making them practical to share, inspect, or integrate into broader reporting systems. They can be created in headless (i.e. terminal only) environments while being interactively inspected afterwards.

**Availability and implementation:**

Alignoth is freely available under the MIT license at https://github.com/alignoth/alignoth (doi: https://doi.org/10.5281/zenodo.15837719). It is implemented in Rust and can be installed via Cargo or Conda.

## 1 Introduction

Visual inspection of aligned reads (also called pileups) is a critical part of various kinds of DNA or RNA sequencing analyses (Corominas *et al.* 2022). In clinical contexts, e.g. pileups are regularly inspected to validate the presence or absence of candidate variants by eye. Plots of these alignments are often shared among collaborators or used in molecular tumor board preparation to support clinical decision-making. In order to create these plots, various alignment viewers have been developed, which can be roughly categorized into graphical desktop applications, command line tools, and web-based tools. However, they often fall short in one or more dimensions: graphical desktop applications like IGV (Thorvaldsdóttir *et al.* 2013) are powerful but hardly suited for automation and difficult to deploy and run in headless (non-graphical, e.g. cluster or cloud based) environments. Conversely, command-line tools such as samtools tview (Li *et al.* 2009) are easily automated using workflow management systems like Snakemake (Mölder *et al.* 2021) or Nextflow (Di Tommaso *et al.* 2017) but provide only static or text-based views with limited interactivity. Finally, web-based tools usually rely on server-side components and supporting services, which require ongoing maintenance and limit usability in privacy-sensitive or restricted environments.

With *Alignoth*, we present a novel tool that aims to unify the individual strengths of all above mentioned approaches without exhibiting the associated disadvantages: while offering a command line interface for headless (e.g. cluster or cloud) execution and automation via reproducible [e.g. Snakemake (Mölder *et al.* 2021) or Nextflow (Di Tommaso *et al.* 2017)] workflows, it generates highly interactive web-browser based pileup plots that can be viewed, explored and shared without installing specialized software.

## 2 Features

Via a command line interface, *Alignoth* utilizes the Vega-Lite plotting library (Satyanarayan *et al.* 2017) to produce read pileup visualizations from alignment [SAM, BAM, CRAM (Li *et al.* 2009)] and reference (FASTA) data in both static (SVG, PNG, PDF) and interactive (HTML) formats. Additionally, it can output the data (JSON, TSV) underlying the visualizations for further usage (e.g. embedding into downstream applications). The HTML output is self-contained, embedding both data and visualization logic without external dependencies. This makes it practical for privacy-sensitive environments such as hospitals, where remote data hosting or authentication is difficult. At the same time, it remains portable—interactive plots can be shared simply by uploading the HTML file to static hosts like GitHub Pages or sending them via email or messaging services of choice. Since they open directly in any modern web browser, they offer system-independent access across platforms. Because plot generation via CLI and interactive exploration are decoupled, *Alignoth* can be run entirely in headless environments such as computing clusters or cloud-based workflows, making it especially suitable for remote settings without graphical interfaces.

**Figure 1.**
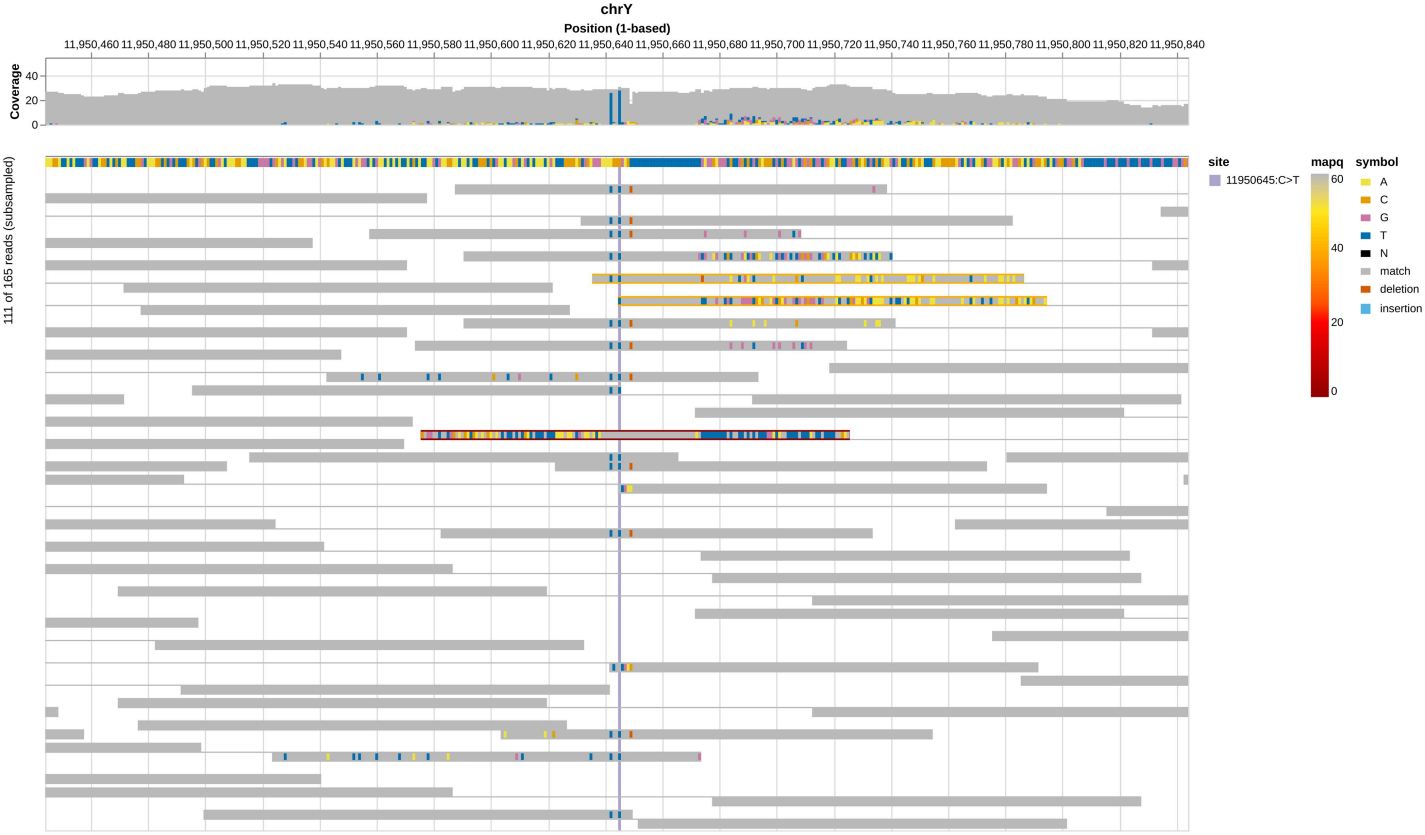
Example alignment visualization generated with Alignoth. The reference sequence together with the read coverage is shown at the top. Matched bases in the aligned reads are shown in grey, while mismatches are color-coded by their nucleotide. Paired-end reads are connected by thin linking segments.

The interactive view supports highlighting specific reads and filtering by read name through a text input field, aiding manual inspection and exploration. While the core layout follows the established IGV-style pileup visualization (Thorvaldsdóttir *et al.* 2013), *Alignoth* introduces additional features aimed at improving interpretability. For example, the border color of each read encodes its mapping quality (Li *et al.* 2009): highly certain read placements (MAPQ ≥60) are shown with grey borders, while uncertain read placements fade through yellow to red, making problematic mappings immediately recognizable (see Fig. 1). All colors used in *Alignoth* follow a colorblind-safe scheme proposed by Wong (2011), ensuring accessibility for all readers.

*Alignoth* ensures compact but non-overlapping read visualization by minimizing the number of needed rows. Let *F* be a set of fragments to be plotted. For each fragment f∈F, let $I_{f}$ denote the interval the fragment covers on the chromosome (from the leftmost aligned base to the rightmost aligned base). The row assignment problem is to find a sequence of rows R=1,2,…,r and an assignment ρ:F→R of each fragment f∈F such that *r* is minimal and there is no pair of overlapping fragments on the same row:


$$\forall f,f^{\prime }\in F:\rho (f)\neq \rho (f^{\prime })\vee I_{f}\cap I_{f^{\prime }}=\emptyset$$

We can show that the row assignment problem is equivalent to an optimal coloring of an interval graph as defined by Olariu (1991). We construct a graph G:=(V,E) with vertices $V:=\{v_{f}|f\in F\}$ and edges


$$(v_{f},v_{g})\in E\;\Leftrightarrow \;I_{f}\cap I_{g}\neq \emptyset .$$

A row assignment then corresponds to a proper vertex coloring, i.e. a function ρ:V→{1,…,r} such that


$$(v_{f},v_{g})\in E\;\Rightarrow \;\rho (v_{f})\neq \rho (v_{g}).$$

The optimal coloring of interval graphs—and thus a row assignment minimizing the number of rows without introducing overlapping fragments—can be obtained in linear time by the greedy color(G) procedure described by Olariu (1991).

**Figure 2.**
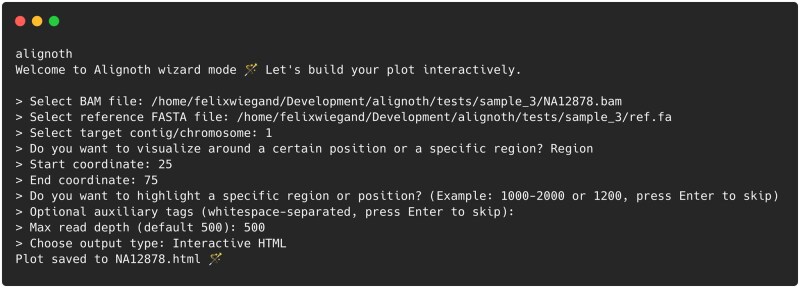
Example of the interactive wizard mode in *Alignoth*. Users are guided step-by-step to generate an alignment plot by specifying input files, genomic region of interest, optional annotations, and output format. If invoked in a directory containing BAM and FASTA files, *Alignoth* automatically detects them via their suffix and provides toggle menus for file selection.

To facilitate integration into automated workflows, *Alignoth* operates via a single command-line interface. Required inputs are a BAM file, a reference genome in FASTA format, and a genomic region (e.g. 17:7661779–7687546) to visualize. Optional arguments allow for flexible customization, including automatic window selection around a base position (--around), read subsampling [--plot-all for plotting all reads, --max-read-depth for subsampling (subsampling is random but uses a fixed seed for reproducibility)], and display of auxiliary alignment tags in tooltips (--aux-tag). In addition, *Alignoth* can highlight different genomic regions or variant sites using the --highlight (explicitly specified regions such as e.g. BRCA1:41196311–41277500, where *BRCA1* serves as an arbitrary example label), --vcf (variant sites automatically inferred from a VCF or BCF file), or --bed (regions inferred from a BED file) parameters. *Alignoth* can also export the Vega-Lite specification along with read, reference, and highlight data in JSON or TSV format, allowing for even more flexible usage—e.g. embedding customized plots directly in Datavzrd reports (Wiegand *et al.* 2025). Furthermore, a dedicated Snakemake (Mölder *et al.* 2021) *meta-wrapper* (https://snakemake-wrappers.readthedocs.io/en/stable/meta-wrappers/bio/alignoth_report.html), that accepts a combination of variant, alignment and reference files, uses Vembrane (Hartmann *et al.* 2023) to convert the variant file into a TSV table suitable for Datavzrd and prepares a full Datavzrd report, including a per-variant overview table with linkouts to an *Alignoth* alignment view for a 1000 bp region around the variant in question. An example report, hosted via GitHub pages, is available under https://alignoth.github.io/alignoth-example-report. To simplify ad hoc usage, *Alignoth* offers an interactive (but still non-graphical) mode that launches a guided prompt-based wizard when run without arguments, allowing users to build plots step-by-step without writing a single command (cf. Fig. 2). This makes *Alignoth* suitable for diverse use cases ranging from pipeline-integrated QC reporting to manual review of variant evidence.

To minimize the size of the resulting HTML files while preserving all relevant alignment information, *Alignoth* uses a compact encoding scheme for read features. We compute a representation of each read in Rust, using rust-htslib (https://github.com/rust-bio/rust-htslib) for parsing BAM files and rust-bio (Köster 2016) for fast reference sequence access. Each alignment is encoded as a CIGAR-like (https://samtools.github.io/hts-specs/SAMv1.pdf) (Li *et al.* 2009) string (e.g. 50=|3d|10=|1C|1G|iGGT) that captures matches, mismatches, insertions, and deletions in a minimal form. In other words, we only store edit operations relative to the reference sequence instead of each actual read sequence. This format is parsed at runtime using Vega-Lite’s transform mechanism (https://vega.github.io/vega-lite/docs/transform.html) to split individual alignment elements for visualization. To further reduce redundancy, we concatenate all required per-read metadata (position, mapping quality, and identifier) into a single whitespace-delimited string (note that it is not possible to provide true binary data in static HTML files) and join all reads using a sentinel delimiter (§). This results in a flat, compact “values” string in the JSON specification, which is decoded entirely client-side. Finally, to minimize the overall size of the Vega-Lite specifications embedded in the HTML files, *Alignoth* compresses them via lz-string (https://pieroxy.net/blog/pages/lz-string/index.html), a JavaScript library implementing Lempel–Ziv compression while ensuring the result remains a valid JavaScript string.

## 3 Results

**Figure 3.**
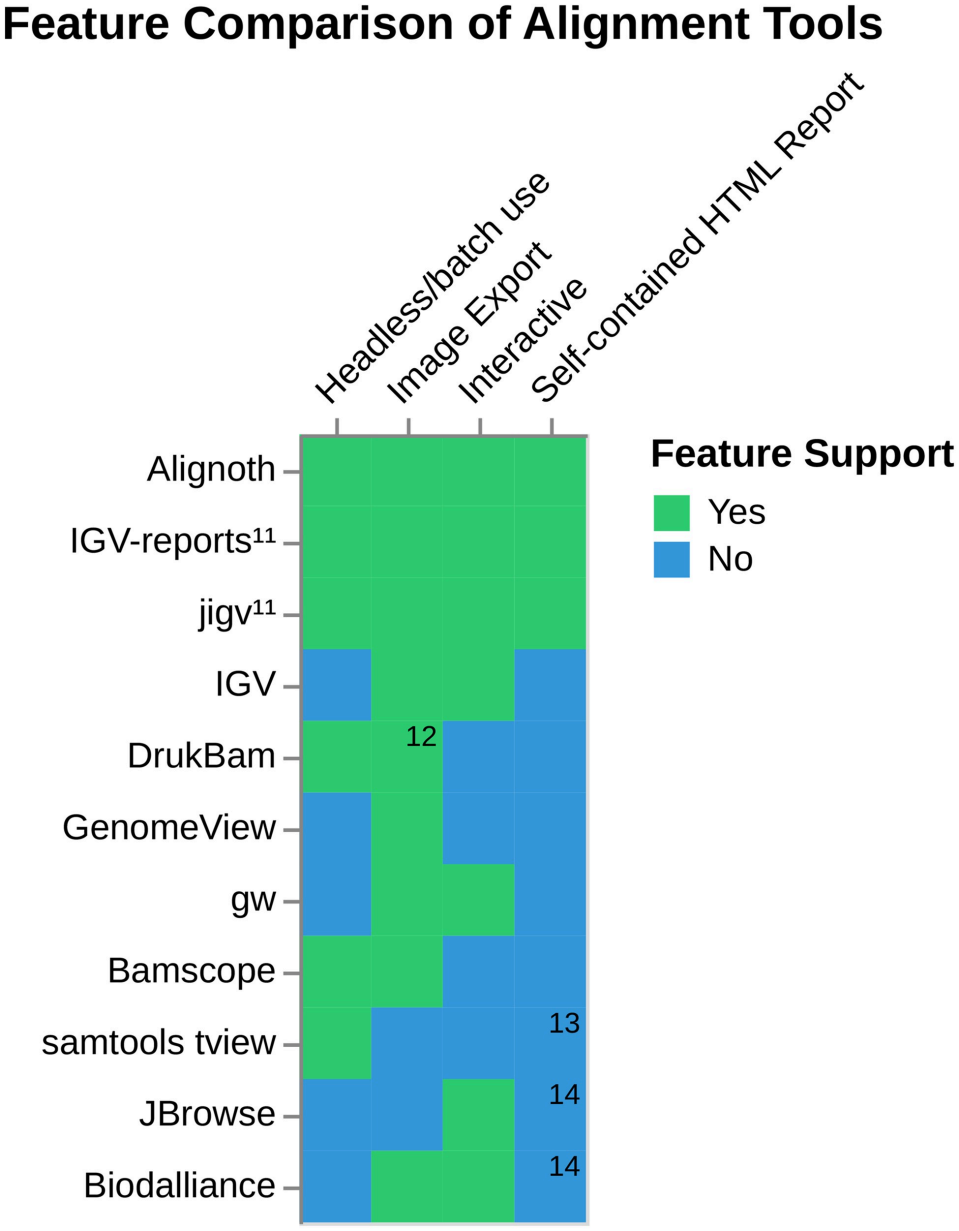
Comparison of read alignment visualization tools. Only Alignoth, jigv and IGV-reports offer self-contained, interactive HTML reports. Headless/batch use refers to the ability to generate visualizations without a graphical user interface, enabling usage in HPC clusters or automated workflows. The list of considered features is restricted to those relevant for this publication. There are various additional features offered by each listed tool. Features are listed as present at the time of publication, please check the individual tools for updates. 11: Requires genomic sites (e.g. from VCF) 12: supports PNG only 13: although samtools tview offers an HTML output mode, it only renders plain-text views with basic color highlighting and lacks interactivity. We therefore do not consider it a full-featured HTML report 14: built with HTML and JavaScript but requires a separate server process. As it does not produce standalone files, we do not consider it a self-contained HTML report

A comparison of existing alignment visualization tools in terms of portability, interactivity, image export, and integration into analysis workflows is provided in Fig. 3. Beyond this comparison from the perspective of *Alignoth*, we are certain that each of these tools provides a considerable value to its respective user group, and encourage the reader to draw their own conclusions based on personal needs and experience.

Among those, the only tools providing a comparable set of features are IGV-reports (https://github.com/igvteam/igv-reports) and jigv (github.com/brentp/jigv). However, there are important architectural and practical differences. *Alignoth* performs explicit read-to-reference alignment processing at plot generation time, whereas IGV-reports and jigv defer this to the client browser by embedding raw alignment data (BAM, FASTA, and VCF) directly using a base64 encoding and render it using IGV.js (Robinson *et al.* 2023). We analyze the implied differences in report generation and browser rendering runtime as well as disk footprint below.

**Figure 4.**
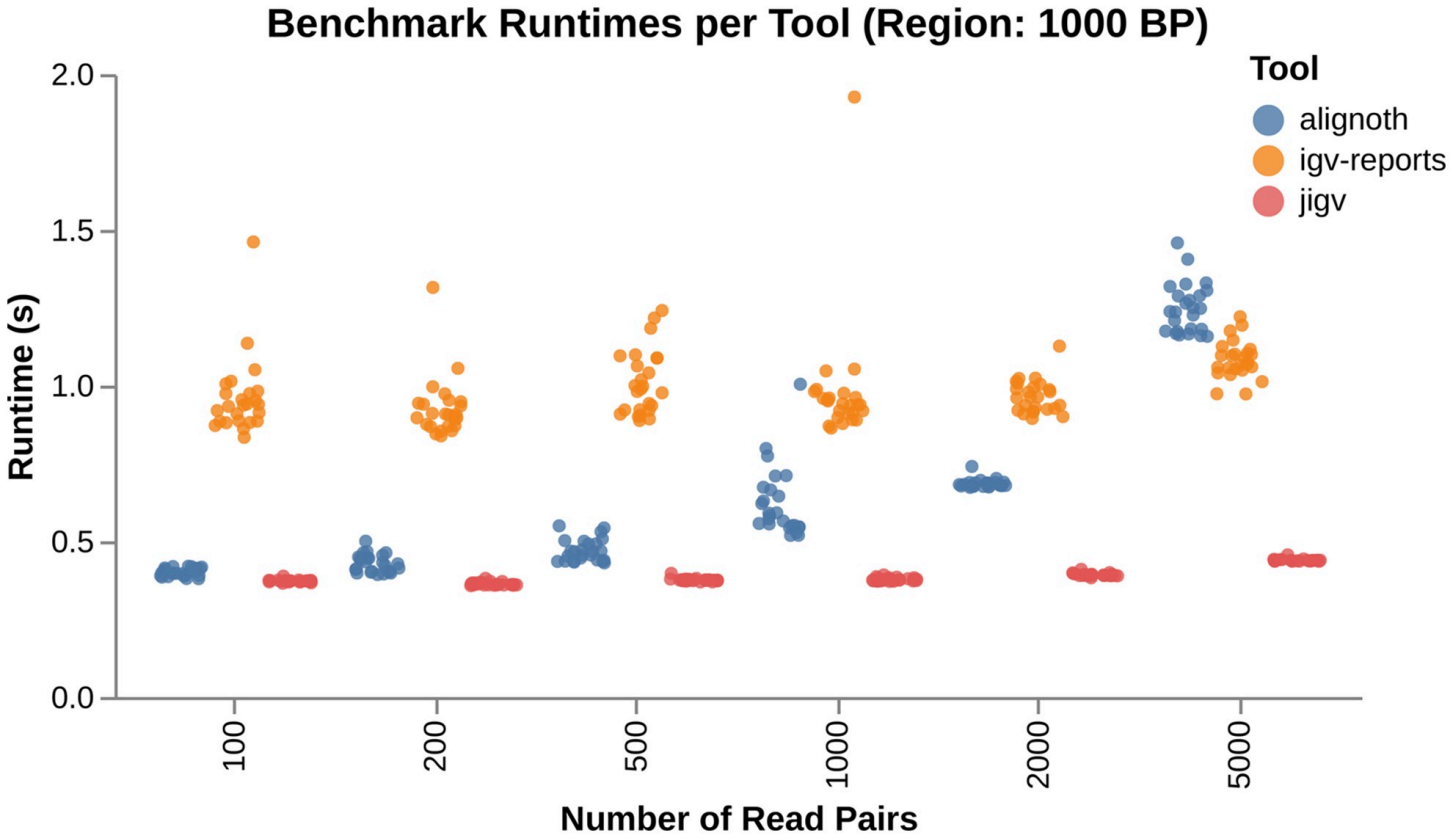
Runtime comparison between *Alignoth*, IGV-reports and jigv for generating alignment plots at varying read depths.

*Alignoth* matches jigv and outperforms IGV-reports in report generation runtime for small datasets (e.g. 100, 200, 500 read pairs; see Fig. 4). For larger datasets both IGV-reports and jigv provide faster report generation.

All tools render comparably fast in the browser: for a test dataset with 1000 reads, final rendering and layout completion occurred at 1.09 s for *Alignoth*, 1.16 s for IGV-reports and 1.48 s for jigv (measured using Safari’s timeline profiling).

**Figure 5.**
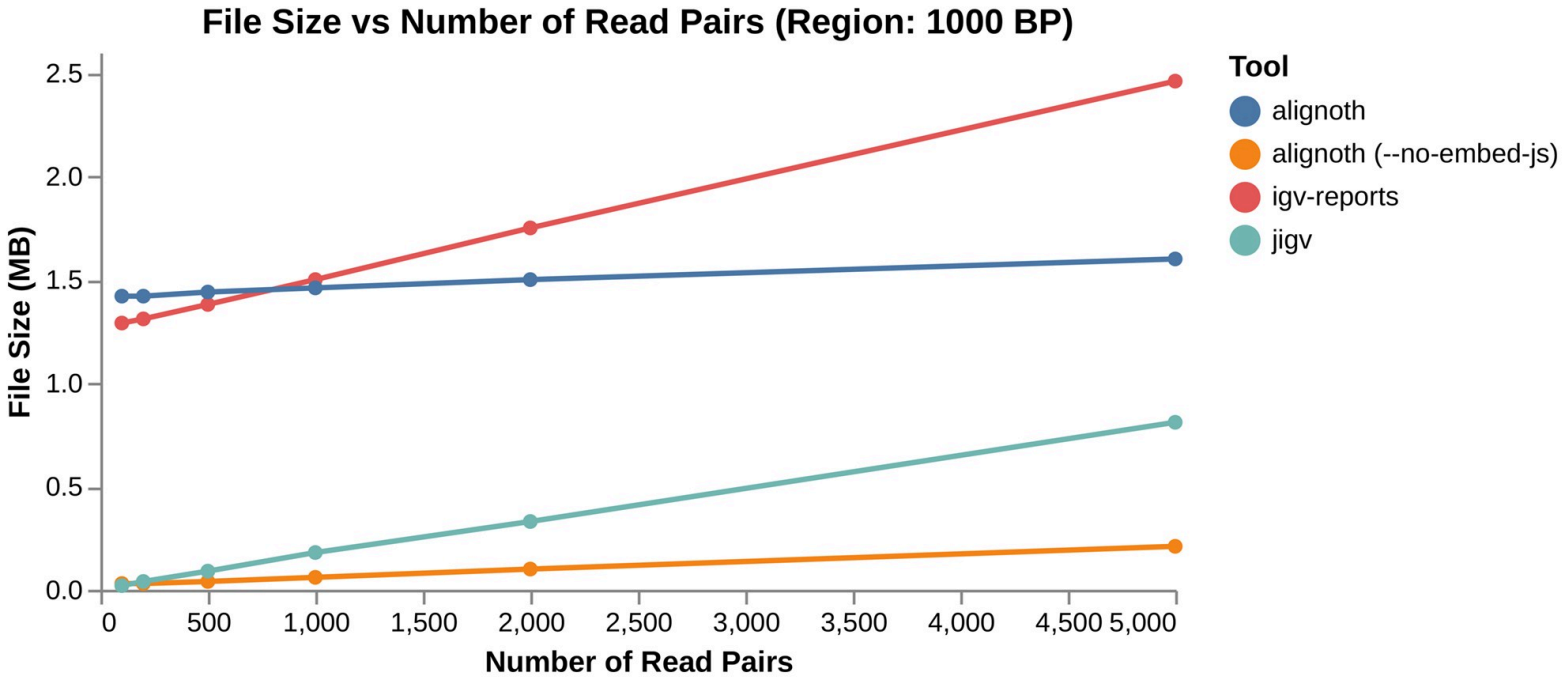
Comparison of output file sizes for interactive HTML alignment reports across varying read depths for a 1000 bp region. *Alignoth* and IGV-reports produce self-contained files with embedded JavaScript dependencies, while jigv and *Alignoth* with --no-embed-js fetch dependencies from external CDNs.

As shown in Fig. 5, *Alignoth* and IGV-reports produce similarly sized self-contained HTML files for small datasets, both embedding JavaScript dependencies for offline use. In contrast, jigv fetches dependencies from external CDNs, requiring internet access when opening a report but producing smaller files. For direct comparison, we also evaluated *Alignoth* with the --no-embed-js flag, which mimics jigv’s behavior and yields similarly sized reports for small datasets. As the number of reads increases, *Alignoth* scales substantially better than both jigv and IGV-reports, producing more storage-efficient files whether or not JavaScript dependencies are embedded.

Unlike *Alignoth*, IGV-reports and jigv require a list of genomic sites (e.g. provided via VCF, BED, BEDPE, or tab-delimited files)—even when no variant annotations are of interest (for benchmarking, we supplied a minimal single-site file to enable a fair comparison). *Alignoth*, by contrast, instead allows to highlight regions of interest (including e.g. variants) via the --highlight option or infers highlighted regions via optionally provided BED (--bed) or VCF (--vcf) files.

## 4 Conclusion

Here, we presented *Alignoth*, a flexible and lightweight tool for generating alignment visualizations directly from BAM files. Its flexible output formats enable both automated reporting and interactive exploration, without compromising platform independence or portability. By combining ease of use with automation via the command line and a small disk footprint, *Alignoth* fills a practical gap in the current ecosystem of alignment visualization tools.

## Data Availability

*Alignoth* is implemented as a command line application with the *Rust* (https://www.rust-lang.org) programming language. It is available as an MIT licensed open source software via *Github* (https://github.com/alignoth/alignoth) (doi: https://doi.org/10.5281/zenodo.15837719), and can be installed via *Bioconda* (https://anaconda.org/bioconda/alignoth) (Grüning *et al.* 2018). The workflow used to evaluate *Alignoth* is available via *Github* (https://github.com/koesterlab/alignoth-benchmark) (doi: https://doi.org/10.5281/zenodo.17669519).
